# Safety of intraocular anti-VEGF antibody treatment under *in vitro* HTLV-1 infection

**DOI:** 10.3389/fimmu.2022.1089286

**Published:** 2023-01-25

**Authors:** Yuan Zong, Koju Kamoi, Hisako Kurozumi-Karube, Jing Zhang, Mingming Yang, Kyoko Ohno-Matsui

**Affiliations:** Department of Ophthalmology & Visual Science, Graduate School of Medical and Dental Sciences, Tokyo Medical and Dental University, Tokyo, Japan

**Keywords:** human T-cell leukemia virus type 1, HTLV-1 uveitis, ocular inflammation, uveitis, aflibercept, VEGF

## Abstract

**Introduction:**

HTLV-1 (human T-cell lymphotropic virus type 1) is a retrovirus that infects approximately 20 million people worldwide. Many diseases are caused by this virus, including HTLV-1–associated myelopathy, adult T-cell leukemia, and HTLV-1 uveitis. Intraocular anti–vascular endothelial growth factor (VEGF) antibody injection has been widely used in ophthalmology, and it is reportedly effective against age-related macular degeneration, complications of diabetic retinopathy, and retinal vein occlusions. HTLV-1 mimics VEGF_165_, the predominant isoform of VEGF, to recruit neuropilin-1 and heparan sulfate proteoglycans. VEGF_165_ is also a selective competitor of HTLV-1 entry. Here, we investigated the effects of an anti-VEGF antibody on ocular status under conditions of HTLV-1 infection *in vitro*.

**Methods:**

We used MT2 and TL-Om1 cells as HTLV-1–infected cells and Jurkat cells as controls. Primary human retinal pigment epithelial cells (HRPEpiCs) and ARPE19 HRPEpiCs were used as ocular cells; MT2/TL-Om1/Jurkat cells and HRPEpiCs/ARPE19 cells were co-cultured to simulate the intraocular environment of HTLV-1–infected patients. Aflibercept was administered as an anti-VEGF antibody. To avoid possible T-cell adhesion, we lethally irradiated MT2/TL-Om1/Jurkat cells prior to the experiments.

**Results:**

Anti-VEGF antibody treatment had no effect on activated NF-κB production, inflammatory cytokines, chemokines, HTLV-1 proviral load (PVL), or cell counts in the retinal pigment epithelium (RPE) under MT2 co-culture conditions. Under TL-Om1 co-culture conditions, anti-VEGF antibody treatment did not affect the production of activated NF-κB, chemokines, PVL, or cell counts, but production of the inflammatory cytokine IL-6 was increased. In addition, anti-VEGF treatment did not affect PVL in HTLV-1–infected T cells.

**Conclusion:**

This preliminary *in vitro* assessment indicates that intraocular anti-VEGF antibody treatment for HTLV-1 infection does not exacerbate HTLV-1–related inflammation and thus may be safe for use.

## Introduction

Human T-cell leukemia virus type 1 (HTLV-1), also known as human T-lymphotropic virus type 1, was the first retrovirus identified as infecting humans and causing disease (1–7). There have been reports of HTLV-1 infection throughout the world, but it is highly endemic in southwestern Japan and in central Australia, sub-Saharan Africa, South America, and the Middle East (8–11). HTLV-1 received global attention after over 40% of aboriginal people in central Australia were confirmed to be infected with the virus (12, 13). Numerous studies have demonstrated the potential impact of this virus, thus attracting the attention of the World Health Organization and many medical experts (14–16). A recent meta-analysis reviewing 3318 relevant studies found that patients with HTLV-1 had a higher adjusted risk of death from any cause compared with HTLV-1 negative controls (RR 1.57, 95% CI 1.37–1.80). In addition, 16 other diseases, including seborrheic dermatitis and Sjogren’s syndrome, were significantly associated with HTLV-1 infection or a greatly increased risk of HTLV-1 infection (17). Furthermore, a recent Japanese study examining data from seroconverted blood donors to estimate new HTLV-1 infections found that increased frequency of sexual contact without condom use led to more widespread horizontal transmission of HTLV-1, resulting in a concerning significant increase in HTLV-1 infections in adolescents and young adults (14, 18).

HTLV-1 causes many diseases, including hematological neoplasms such as adult T-cell leukemia/lymphoma (ATL) and inflammatory diseases such as HTLV-1 uveitis (HU) and HTLV-1–associated myelopathy (3–5, 7, 19, 20). HU is a form of intermediate uveitis that requires ophthalmic care (21–24). In regions of high HTLV-1 prevalence, HU is currently the most common form of uveitis (25, 26). Recent studies of HU have revealed the capability of horizontal transmission (27, 28) and onset of disease at low proviral load (PVL) in patients with Grave’s disease (29, 30). HTLV-1 is also associated with ATL-related ocular manifestations (19, 31, 32). In a nationwide survey conducted in Japan, ocular infiltration by HTLV-1–infected cells was found to be the most common symptom of HTLV-1–associated ATL (33).

Vascular endothelial growth factor (VEGF) along with VEGF receptors (VEGFRs), are crucial for angiogenesis, vascular permeability, and endothelial cell proliferation (34, 35). Ophthalmologically, VEGF plays a central role in age-related macular degeneration (AMD), retinal vein occlusions, and complications of diabetic retinopathy. Anti-VEGF drugs administered *via* intravitreal injection have been shown to be remarkably effective in treating the abovementioned diseases (36, 37). In recent years, anti-VEGF therapies have been increasingly utilized in ophthalmology. A study conducted in the capital region of Denmark reported that a total of 3684 AMD patients were treated with anti-VEGF therapy in 2019, compared to 576 patients in 2007 (38). Aflibercept (EYLEA^®^), a 115-kDa recombinant fusion decoy protein, is a representative intraocular anti-VEGF drug. Aflibercept consists of the VEGF-binding domains of human VEGFR-1 and VEGFR-2 fused to the Fc domain of human immunoglobulin G1 (39). The drug binds all forms of VEGF-A as well as another member of the VEGF family, placenta growth factor, with higher affinity than other anti-VEGF antibodies (40–42).

**Figure 1:**
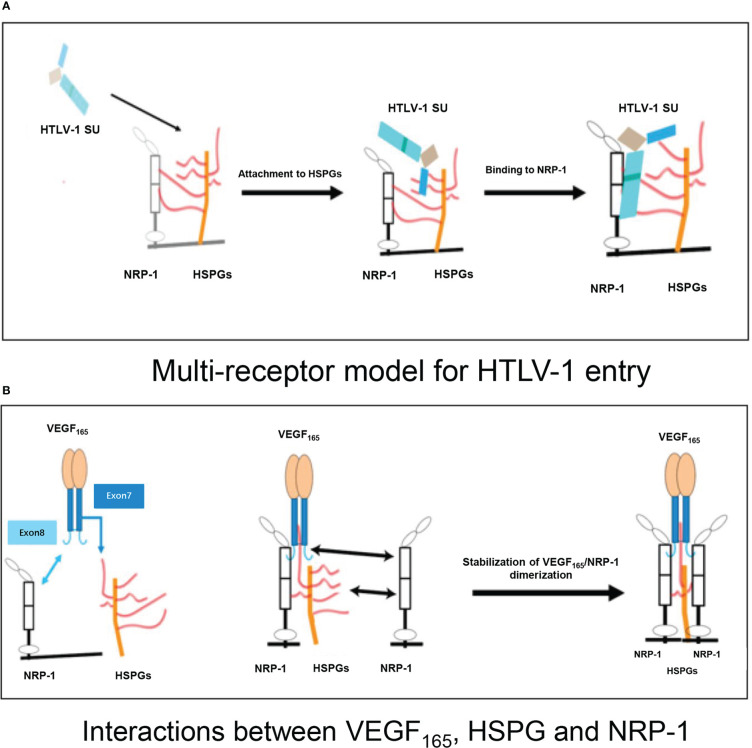
**(A)** Multi-receptor model of the initial phase of HTLV-1 entry into target cells. HTLV-1 SU interacts with HSPG, resulting in the initial attachment and concentration of HTLV-1 particles on the cell surface. The interaction of HSPG with SU and NRP-1, and the direct binding of SU to NRP-1 then result in the recruitment of NRP-1, enabling stable binding of SU to the HSPG/NRP-1 complex. **(B)** Schematic representation of interactions between VEGF_165_, HSPG, and NRP-1. The sequence encoded by exon 7 of the VEGF_165_ gene binds to HSPG, and the sequence encoded by exon 8 binds directly to NRP-1. The NRP-1 dimer is then formed, resulting in enhanced stability. HTLV-1: human T-cell lymphotropic virus type 1; SU: surface subunit; HSPG: heparan sulfate proteoglycan; NRP-1: neuropilin-1.

HTLV-1 exploits heparan sulfate proteoglycans (HSPGs) to gain entry into CD4+ T cells *via* molecular mimicry of VEGF_165_, with initial association with neuropilin-1 (NRP-1) (**Figure 1A**) (43–45). VEGF_165_ is the predominant isoform of VEGF (35, 46), and its triggering of signal transduction first requires the mediation of NRP-1 with HSPG (**Figure 1B**) (45, 47). Previously reported evidence indicates that HTLV-1 entry is dramatically reduced in cells treated with either VEGF_165_ exon 7– or exon 8–like peptides (45).

Despite the strong relationship between VEGF and HTLV-1 infection, safety studies have not been conducted on anti-VEGF antibodies for intraocular use in HTLV-1 carriers. Considering the large number of individuals infected with HTLV-1 and the promising application of anti-VEGF treatments for ocular neovascularization–related diseases such as AMD, the absence of such studies has undoubtedly increased the likelihood of HTLV-1–associated ocular diseases occurring in HTLV-1 carriers who could benefit from anti-VEGF therapy. Therefore, this *in vitro* study investigated the effect of anti-VEGF antibody treatment on the eye under conditions of HTLV-1 infection using an ocular cell line and HTLV-1–infected T-cell lines (24, 48) and the maintenance of immunological homeostasis in the eye (48, 49). VEGF can exacerbate disruption of the blood-retinal barrier by promoting choroidal vascular invasion (50). Therefore, the human retinal pigment epithelial cell line ARPE19 and primary human retinal pigment epithelial cells (HRPEpiCs) were chosen as ocular cells for this study.

## Materials and methods

### Cell culture

ARPE19 cells (American Type Culture Collection, Manassas, VA) were cultured in DMEM (Invitrogen) supplemented with 1% penicillin/streptomycin as well as 10% fetal bovine serum (Invitrogen). HRPEpiCs (ScienceCell Research Laboratories, Carlsbad, CA) were originally isolated from a human retina and grown in this study in epithelial cell culture medium (ScienceCell Research Laboratories) consisting of 5% fetal bovine serum, 1% penicillin/streptomycin, and 1% epithelial cell growth supplement. HRPEpiCs were used within the first four passages. The MT2 and TL-Om1 cell lines were used as HTLV-1–infected T cells, and Jurkat cells were used as control T cells (51, 52). MT2, TL-Om1, and Jurkat cells were cultured in RPMI 1640 medium (Wako Pure Chemical Corp.) with 10% fetal bovine serum (Invitrogen) and 1% penicillin/streptomycin. All cells were incubated at 37°C under 5% CO_2_. In all experiments except cytometric bead assays, T cells used for co-culture were irradiated with 9000 rads before the experiment.

### VEGF inhibitor

Aflibercept (EYLEA^®^; Santen Pharmaceutical, Osaka, Japan) was used as a VEGF inhibitor. Based on previous studies (53, 54), the concentration of aflibercept used in this study was 0.5 mg/mL of culture medium, equivalent to the intraocular clinical dose of aflibercept after considering presumed vitreous dilution.

### Cell co-culture

In this study, the standard *in vitro* HTLV-1 infection method was employed (51, 55, 56). Briefly, 1.5 × 10^5^ HRPEpiCs or ARPE19 cells were plated and co-cultured for 48 h with or without aflibercept with triple the number of irradiated (9000 rads) MT2, TL-Om1, or Jurkat cells in 24-well plates. To confirm the absence of the abovementioned HTLV-1–infected cell lines, MT2/TL-Om1/Jurkat cells were removed and then the attached HRPEpiCs were washed and passaged every 2 days for a total of three passages.

### NF-κB activity ELISA

In ARPE19 cells co-cultured with HTLV-1–infected cell lines, the effects of aflibercept treatment were measured using an InstantOne ELISA kit (Cat. No. 85–86083–11. eBioscience, San Diego, CA) in accordance with the manufacturer’s instructions. Absorbance was measured at a wavelength of 450 nm.

### HTLV-1 PVL measurement

MT2 or TL-Om1 cells were cultured in RPMI 1640 medium containing 10% fetal bovine serum and 1% penicillin/streptomycin with 0.5 mg/mL aflibercept. An EZ1 Virus Mini kit v2.0 (Qiagen, Hilden, Germany) was used to prepare DNA from each sample in accordance with the manufacturer’s instructions. To measure HTLV-1 PVL in cells, quantitative real-time polymerase chain reaction (PCR) was used, as previously described (57–59). HTLV-1 Tax primer was used to determine the PVL (forward, 5’-CCCACTTCC CAGGGTTTGGA-3’; reverse, 5’-GGCCAGTAGGGCGTGA-3’) and probe (5’-FAM-CCAGTCTACGTGTTTGGA GACTGTGTACA-TAMRA-3’). Glyceraldehyde-3-phosphate dehydrogenase was used as an internal control. In the same manner, the HTLV-1 PVL in HRPEpiCs co-cultured with HTLV-1–infected cell lines was also measured.

### Cytometric bead array assay

In the case of cytometric bead assays, HRPEpiCs (1.5 × 10^5^ cells/mL) were first allowed to adhere to the wells of 6-well plates overnight. MT2/TL-Om1/Jurkat (5 × 10^5^ cells/mL) cells were then seeded in the wells and co-cultivated with the HRPEpiCs for 48 h. We used the co-culture supernatants and a CBA human inflammatory cytokine kit (BD Bioscience, San Jose, CA) for CBA assays. Based on the instructions provided by the manufacturer, the data were analyzed using FCAP Array software, version 3.0 (BD Bioscience). The cytokines measured included IL-12p70, IL-10, IL-8, IL-6, IL-1β, IFN-γ, and TNF, and the chemokines measured included CXCL10, CXCL9, CCL5, and CCL2. Minimum detection limits for cytokines/chemokines were as follows: IL-1β, 7.2 pg/mL; IL-12p70, 1.9 pg/mL; IL-6, 2.5 pg/mL; IL-8, 0.2 pg/mL; IL-10, 3.3 pg/mL; IFN-γ, 10 pg/mL; TNF, 3.7 pg/mL; CXCL9, 2.5 pg/mL; CXCL10, 2.8 pg/mL; CCL5, 1.0 pg/mL; and CCL2, 2.7 pg/mL.

## Results

### Effect of aflibercept on NF-κB activation in ARPE19 cells co-cultured with HTLV-1–infected cell lines

The transcription factor NF-kB is closely associated with both the regulation of VEGF secretion by the RPE and the activation of gene transcription in host cells by HTLV-1 (60, 61). Therefore, to characterize the effect of anti-VEGF treatment on NF-κB activity, in this study, we quantitatively estimated the expression of phospho p65 NF-κB in ARPE19 cells cultured alone or co-cultured with MT2/TL-Om1/Jurkat cells by ELISA.

**Figure 2:**
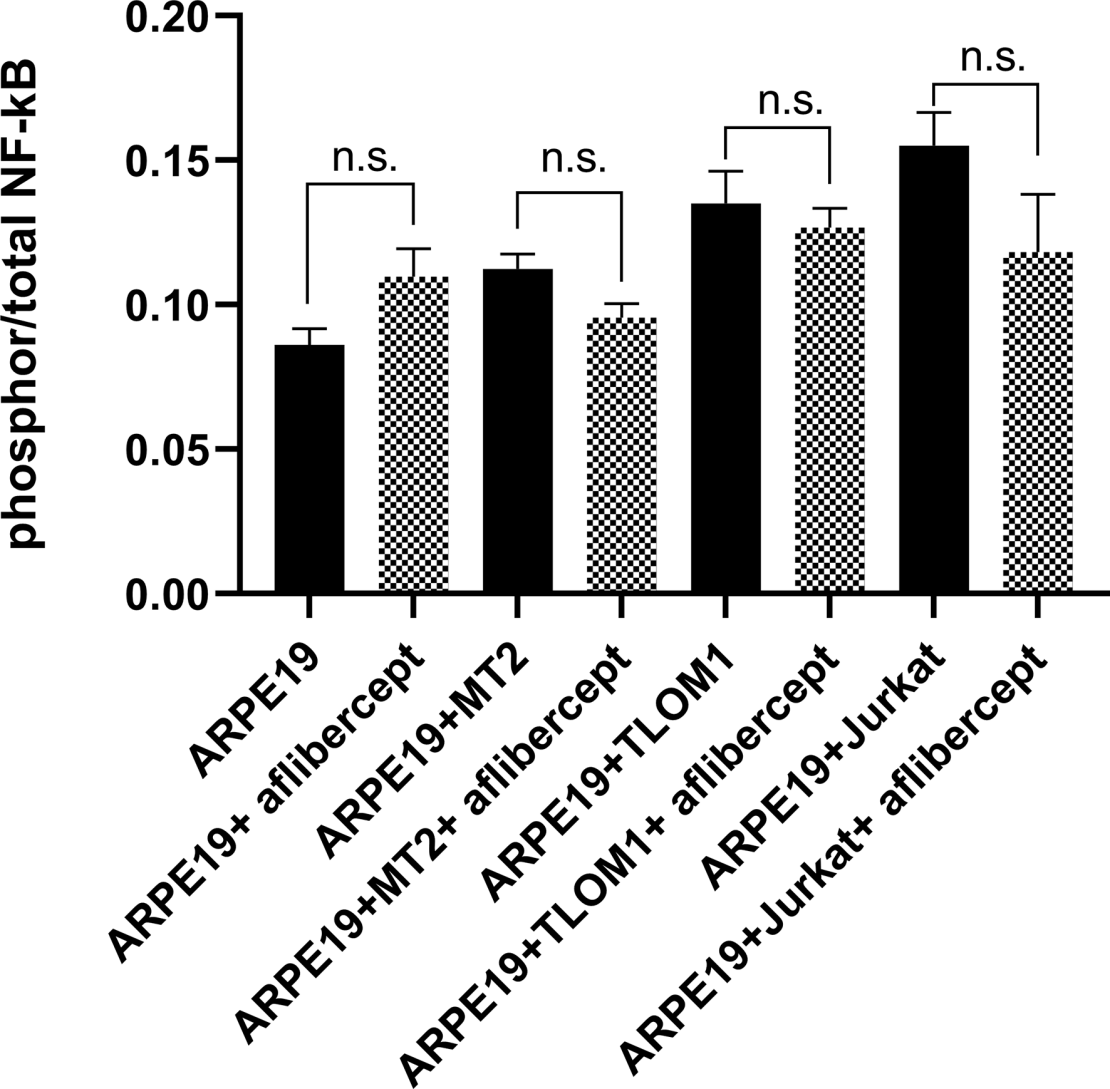
ELISA for phospho p65 NF-κB. Effect of anti-VEGF treatment on activation of nuclear factor-κB (NF-κB) in ARPE19 cells shown as the ratio of phosphor to total. No significant change in NF-κB activation of ARPE19 cells in corresponding groups was seen following aflibercept addition. Data are taken from three independent biological experiments and presented as the mean ± SEM (n.s., not significant).

As described in the Materials and Methods section, prior to all analyses of ARPE19 cells/HRPEpiCs co-cultured with T cell lines, the T cells were irradiated with 9000 rads, a level reportedly lethal for all treated cells (55). Trypan blue staining of the irradiated T cells confirmed that they were not viable 10 days after irradiation. By repeatedly washing the ARPE19 cell/HRPEpiC culture, it was ensured that no irradiated T cells were present at the time measurements were conducted. As shown in **Figure 2**, there was no significant change in mean NF-κB phosphorylation in ARPE19 cells cultured alone or co-cultured with the three T-cell lines after adding 0.5 mg/mL of aflibercept. These results suggest that anti-VEGF treatment does not enhance the NF-κB activation level in ARPE19 cells co-cultured with HTLV-1–infected cell lines.

### Changes in cytokine and chemokine expression in HRPEpiCs co-cultured with HTLV-1–infected T-cell lines treated with aflibercept

Cytokine and chemokine levels in the culture supernatant of HRPEpiCs co-cultured with MT2, TL-Om1, or Jurkat cells and in the supernatant of HRPEpiCs cultured alone were measured at 48 h with/without the addition of aflibercept.

**Figure 3:**
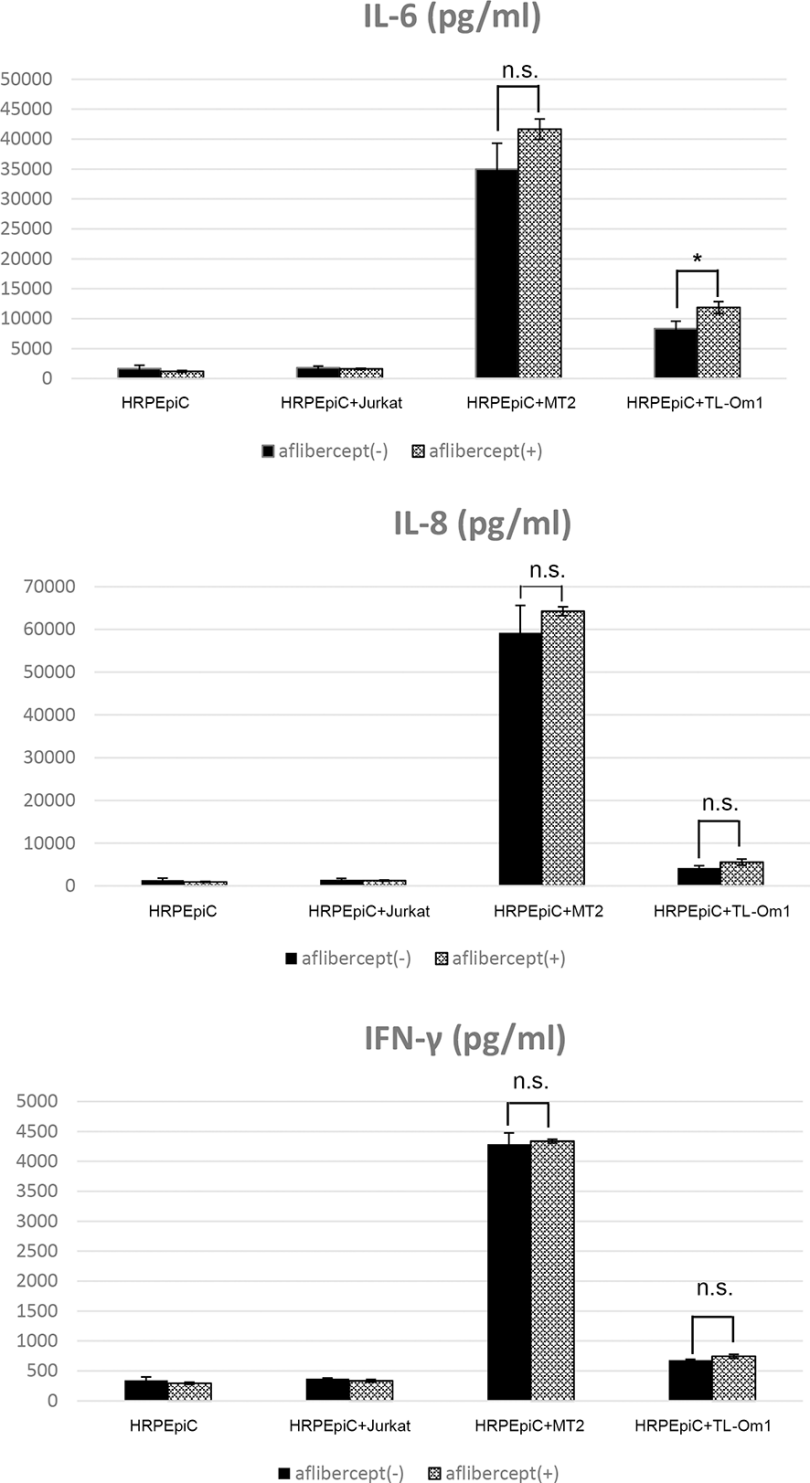
Levels of inflammatory cytokines were measured in the culture supernatants of HRPEpiCs and HRPEpiCs co-cultured for 48 h with MT2, TL-Om1, or Jurkat cells with/without 0.5 mg/mL aflibercept. In co-culture of HRPEpiCs with MT2 cells, aflibercept did not significantly affect the levels of IL-6, IL-8, and IFN-γ. In co-culture of HRPEpiCs with TL-Om1 cells, production of IL-6 increased significantly following aflibercept addition, but levels of IL-8 and IFN-γ did not change significantly. Data are taken from three independent biological experiments and presented as the mean ± SEM (units: pg/μL) (*P < 0.05; n.s., not significant).

When HRPEpiCs were co-cultured with MT2 or TL-Om1 cells, secretion of the inflammatory cytokines IL-6, IL-8, and IFN-γ increased significantly. In HRPEpiCs co-cultured with MT2 or TL-Om1 cells, there were no changes in the levels of IL-8 and IFN-γ after the addition of aflibercept. IL-6 secretion by HRPEpiCs co-cultured with TL-Om1 cells was significantly increased, but an insignificant increase in IL-6 secretion was observed in HRPEpiCs co-cultured with MT2 cells (**Figure 3**).

**Figure 4:**
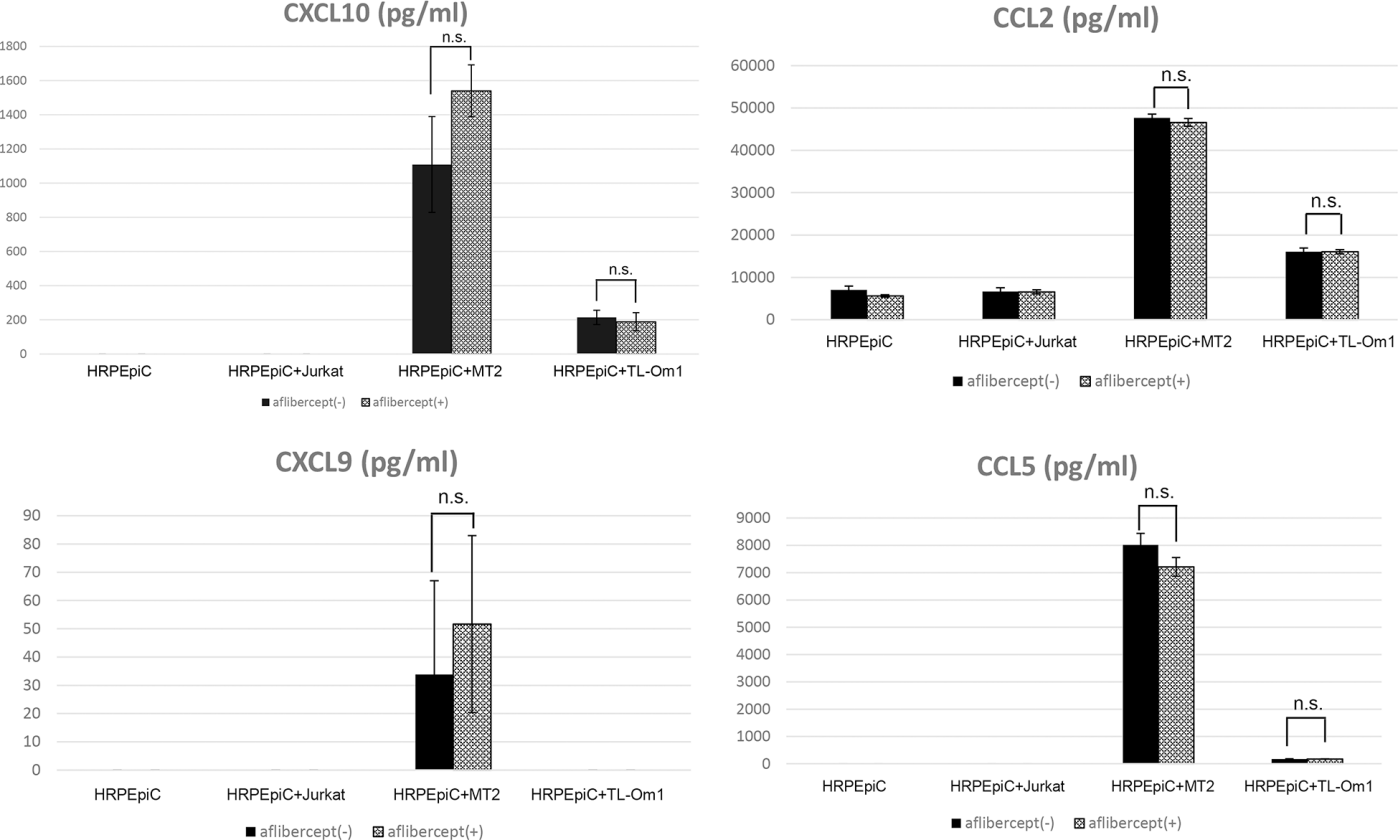
Levels of chemokines measured in the culture supernatants of HRPEpiCs and HRPEpiCs co-cultured for 48 h with MT2, TL-Om1, or Jurkat cells with/without 0.5 mg/mL aflibercept. Secretion of CXCL10, CCL2, CXCL9, and CCL5 was monitored, but no significant differences were detected in all corresponding groups, with or without aflibercept. Data are taken from three independent biological experiments and presented as the mean ± SEM (units: pg/μL) (n.s., not significant).

Secretion of the chemokines CXCL10, CCL2, and CCL5 was significantly increased in the case of HRPEpiC/MT2 cell and HRPEpiC/TL-Om1 cell co-culture. Although CXCL9 secretion was observed in co-culture of HRPEpiCs and MT2 cells, the level was low and variable. In the case of HRPEpiC/TL-Om1 cell co-culture, the level of CXCL9 secretion was below the detection limit. For all chemokines tested, no significant difference in expression level was detected after adding aflibercept (**Figure 4**).

The levels of IL-1β, IL-12p70, IL-10, and TNF were all below the respective detection limits (data not shown).

### Detection of HTLV-1 proviral DNA in HTLV-1–infected T cells treated with aflibercept

**Figure 5:**
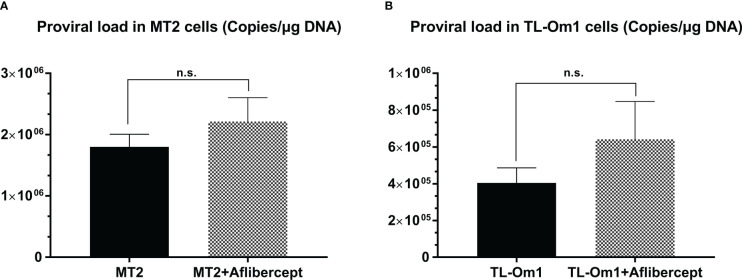
Proviral load (PVL) in MT2 cells **(A)** or TL-Om1 cells **(B)** treated with/without 0.5 mg/mL aflibercept for 48 (h) The number of each type of cells was 5 × 10^5^. Aflibercept had no effect on the PVL of either cell type. Data are taken from three independent biological experiments and presented as the mean ± SEM (n.s., not significant).

The PVL in HTLV-1–infected cells is the most commonly used biomarker for confirmation of the diagnosis and severity of HTLV-1–associated diseases (59, 62). The effect of anti-VEGF treatment on the PVL of MT2 and TL-Om1 cells was measured after 48 h of culture with aflibercept. Mean PVL values of control MT2 and TL-Om1 cells were 1.74 × 10^6^ and 5.34 × 10^5^ copies/μg DNA, respectively, and the mean PVL values of MT2 and TL-Om1 cells treated with aflibercept were 1.51 × 10^6^ and 3.63 × 10^5^ copies/μg DNA, respectively. Aflibercept thus had no significant effect on PVL in these cells (**Figure 5**).

### HTLV-1 proviral DNA detection in HTLV-1–infected HRPEpiCs treated with aflibercept

We measured PVL by real-time PCR to confirm the effect of aflibercept on HTLV-1–related disease progression in HRPEpiCs. As described in the Materials and Methods section, T-cell lines were pre-irradiated with a lethal dose of 9000 rads (55, 56), and 10 days after irradiation, trypan blue staining confirmed the nonviability of irradiated T cells. At the time of DNA isolation, multiple washings ensured that no irradiated T cells remained in the HRPEpiC culture.

**Figure 6:**
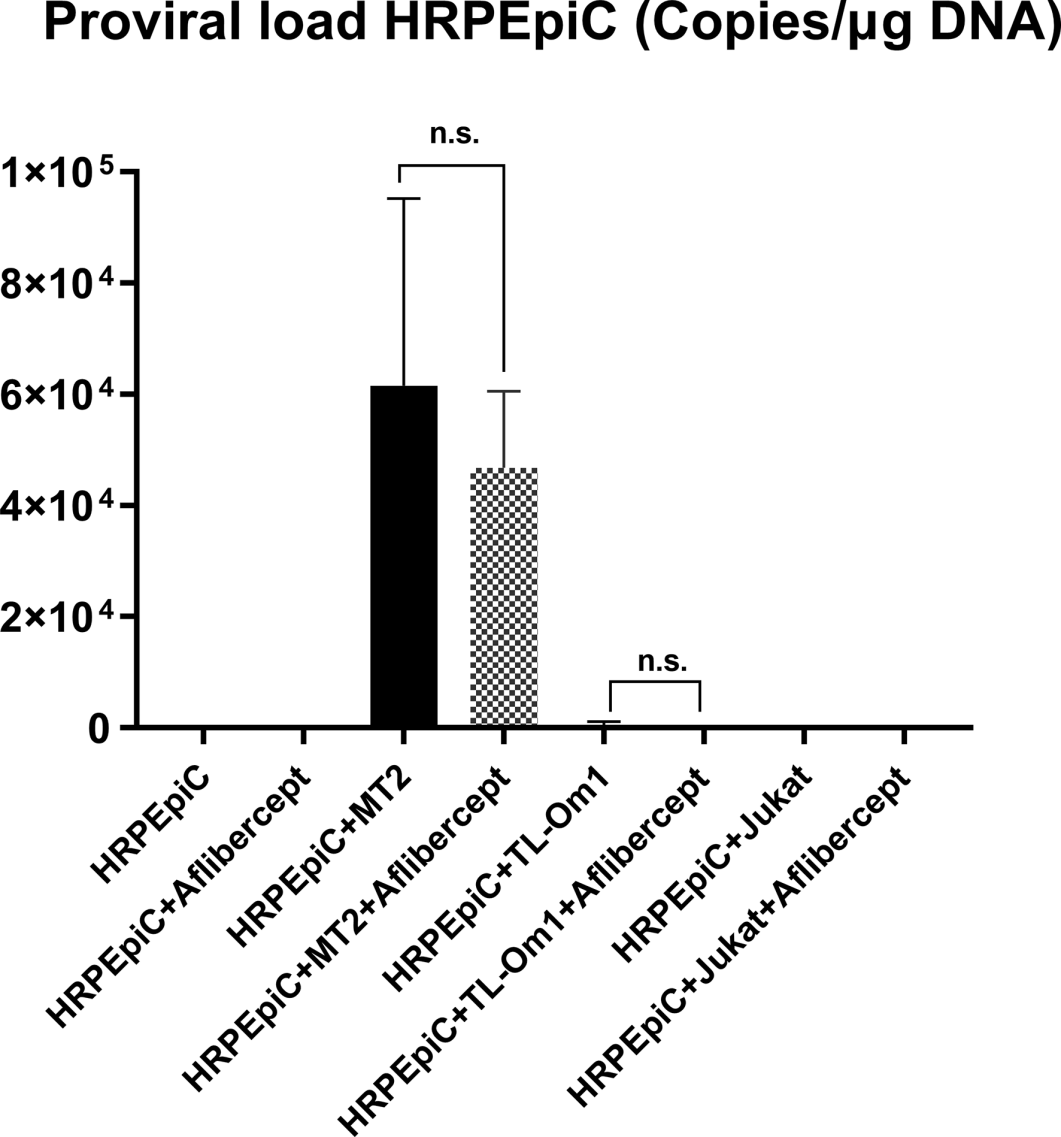
HTLV-1 proviral DNA (PVL) was monitored in HRPEpiCs transferred three times after culture alone or co-culture with MT2, TL-Om1, or Jurkat cells. Aflibercept had no effect on HTLV-1 PVL of HRPEpiCs in the corresponding groups. The number of each type of cells was 1 × 10^5^. Data are taken from three independent biological experiments. Error bars represent standard deviation (n.s., not significant).

As shown in **Figure 6**, no proviral DNA was detected in negative control samples extracted from HRPEpiCs cultured alone or HRPEpiCs co-cultured with irradiated Jurkat cells, with or without aflibercept treatment. Proviral DNA was detected in samples extracted from HRPEpiCs co-cultured with irradiated MT2 cells, indicating that the HRPEpiCs were infected with HTLV-1. The mean PVL in HRPEpiCs co-cultured with MT2 cells not treated with aflibercept was 4.68 × 10^5^ copies/μg DNA. In contrast, the mean PVL in HRPEpiCs co-cultured with MT2 cells treated with aflibercept was 6.15 × 10^5^ copies/μg DNA. Treatment with aflibercept thus had no significant effect on PVL. With regard to HRPEpiCs co-cultured with irradiated TL-Om1 cells, although a very limited amount of proviral DNA (1.65 × 10^3^ copies/μg DNA) was detected in a group of samples without aflibercept, aflibercept had no significant effect on PVL in these cells.

### Effect of aflibercept on the growth of HRPEpiCs and ARPE19 cells

**Figure 7:**
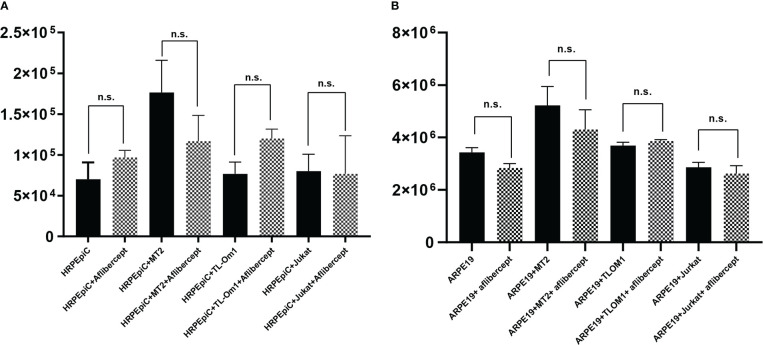
Enumeration of HRPEpiCs **(A)** and ARPE19 cells **(B)** co-cultured with irradiated T-cell lines or cultured alone; data are number of cells per well with/without 0.5 mg/mL aflibercept after three transfers. The number of HRPEpiCs and ARPE19 cells at the beginning of each culture was 1.5 × 10^5^. No significant changes in the number of HRPEpiCs or ARPE19 cells were observed in the corresponding groups with aflibercept addition. Data are taken from three independent biological experiments and presented as the mean ± SEM (n.s., not significant).

To evaluate the effect of aflibercept on cell proliferation, HRPEpiCs/ARPE19 cells co-cultured with irradiated T cells were enumerated after three transfers in order to prevent adhesion of RPE cells to T cells. After three passages, there were no significant changes in the mean cell count of HRPEpiCs cultured alone or co-cultured with MT2/TL-Om1/Jurkat cells after adding 0.5 mg/mL of aflibercept (**Figure 7A**). ARPE19 cells continued to proliferate during the culture process. After three passages, there was also no significant change in the mean number of ARPE19 cells cultured alone or co-cultured with MT2/TL-Om1/Jurkat cells after the addition of aflibercept (**Figure 7B**). These results indicate that under the co-culture conditions examined in this study, aflibercept has no effect on the proliferation of primary or immortalized RPE cells.

## Discussion

Numerous reports have demonstrated the usefulness and efficacy of anti-VEGF therapy for AMD, retinal vein occlusions, and complications of diabetic retinopathy. As a representative drug, aflibercept has been heavily used intraocularly worldwide (37). However, despite the large number of HTLV-1 carriers worldwide, the safety of aflibercept for patients infected with HTLV-1 has not been explored in basic ophthalmologic studies. Numerous studies have demonstrated a relationship between HTLV-1 infection and VEGF (63–66), which is a key factor in the pathogenesis of HU (67). One study reported the development of corneal endotheliitis in a patient infected with human herpesvirus-6 (HHV-6) after intravitreal injection of an anti-VEGF antibody (68). Similar to HTLV-1, HHV-6 is a lymphotropic virus that infects lymphocytes, including CD4^+^ T cells (69–71). The safety of intraocular anti-VEGF treatment in HTLV-1–infected patients is thus of considerable importance. Therefore, we focused on the effects of aflibercept on the eye under conditions of HTLV-1 infection *in vitro* in the present study.

The primary HTLV-1 protein involved in host cell carcinogenesis is Tax, which is also associated with VEGF upregulation (72, 73). The transcription factor NF-kB regulates VEGF secretion in the RPE (60, 74, 75). NF-kB and its family members regulate many genes responsible for immune responses, cell proliferation, apoptosis, and cell migration (76). During HTLV-1–associated disease processes, one of the major mechanisms by which HTLV-1 activates gene transcription in host cells is through regulation of NF-kB activity (61, 77). ARPE19 cells are the most commonly used RPE cell line in retinal biology (78). Therefore, we first investigated whether anti-VEGF treatment alters the phosphorylation activation of NF-kB in ARPE19 cells. No significant change in the phosphorylation activation of NF-kB was observed in any of the ARPE19 cells examined following aflibercept treatment (**Figure 2**), suggesting that anti-VEGF treatment does not alter NF-kB activation in the blood-ocular barrier during HTLV-1 infection.

Having established these data in cell lines, the effect of anti-VEGF antibody treatment on intraocular inflammation in HU patients was evaluated by monitoring changes in cytokine and chemokine expression after co-culture with primary human RPE cells (HRPEpiCs). Cytokines play an important role in virus-induced immunopathology. Studies have shown that levels of the cytokines IL-6 and IL-10 are elevated in the aqueous humor of patients with uveitis. These cytokines play an important role in the pathogenesis and persistence of intraocular inflammation (79, 80). IL-6 is reportedly one of the most measurable inflammatory cytokines in macular edema and thus has predictive value in anti-VEGF treatment (81). A previous animal model study found that by injecting anti-VEGF drugs intravitreally, leukocytes, particularly macrophages, are prevented from infiltrating into the retina. Anti-VEGF can inhibit pre-retinal neovascularization, which is believed to be associated with the secretion of cytokines and chemokines (82, 83). We therefore evaluated the impact of anti-VEGF treatment on the expression of various inflammatory cytokines (IL-12p70, IL-10, IL-8, IL-6, IL-1β, IFN-γ, and TNF) and chemokines (CXCL10, CXCL9, CCL5, and CCL2) under the condition of HTLV-1 infection (i.e., in MT2/TL-Om1 cells in contact with HRPEpiCs).

Our analysis of inflammatory cytokines showed that levels of IL-6, IL-8, and IFN-γ were increased in co-culture of MT2 or TL-Om1 cells with HRPEpiCs (**Figure 2**). These inflammatory cytokines can cause damage to ocular tissues (84, 85). In co-culture of HRPEpiCs and MT2 cells, no significant changes in the production of any inflammatory cytokines were observed after anti-VEGF treatment (**Figure 3**), suggesting that intraocular anti-VEGF treatment of HTLV-1 carriers would neither cause nor aggravate intraocular infections. In co-culture of HRPEpiCs and TL-Om cells, however, expression of the inflammatory cytokine IL-6 was significantly increased following the administration of aflibercept (**Figure 3**), suggesting that aflibercept treatment could induce intraocular inflammation in ATL patients.

Production of the chemokines CXCL10, CCL2, CXCL9, as well as CCL5, was increased during co-culture of MT2 cells and HRPEpiCs, and production of CXCL10, CCL2, and CCL5 was increased during co-culture of TL-Om1 cells and HRPEpiCs (**Figure 4**). An anti-VEGF antibody treatment had no effect on the levels of these chemokines (**Figure 4**). According to these findings, anti-VEGF treatment does not enhance the chemokine-mediated attraction of inflammatory cells to the eye in patients with HTLV-1 infection (86).

In the development of HTLV-1–associated diseases, the PVL of HTLV-1 in the peripheral blood is an important factor (87, 88). It is also a useful biomarker in determining HTLV-1 disease progression (88, 89). Due to the localized concentration in the eye, the risk of inducing HTLV-1–associated inflammation and malignant transformation of ocular cells following aflibercept administration is of concern. VEGF_165_ selectively competes with HTLV-1 for entry into cells (45). Previous investigations identified the RPE as a potential reservoir for HTLV-1, as HTLV-1 proviral DNA was detected in immortalized RPE cells (ARPE19) co-cultured with HTLV-1–infected T cells (55, 58). However, compared with primary cell lines, immortalized cell lines may exhibit abnormal gene expression or biological functions. In contrast, it is likely that isolated primary cultures more accurately reflect *in vivo* cell morphology and function (90). Thus, our study selected primary human retinal pigment epithelial cells (i.e., HRPEpiCs) as representative ocular cells. Using two types of HTLV-1–infected T cells and HRPEpiCs co-cultured with MT2 or TL-Om1 cells, we investigated the effects of anti-VEGF treatment on PVL.

Anti-VEGF treatment had no effect on the PVL of MT2 or TL-Om1 cells, indicating that anti-VEGF treatment did not stimulate transformation of infected T cells (**Figure 5**). To assess the PVL of HRPEpiCs, T cells were pre-exposed to 9000 rads to ensure that the T cells would be completely eliminated during co-culture. We confirmed that no T cells were present after three passages and then determined the HTLV-1 PVL in HRPEpiCs. The presence of HTLV-1 proviral DNA in co-cultured HRPEpiCs was confirmed (**Figure 6**). More importantly, we demonstrated that aflibercept treatment did not increase the PVL in HRPEpiCs (**Figure 6**), indicating that anti-VEGF treatment does not affect PVL in the RPE. These results suggested that aflibercept does not affect HTLV-1 provirus-related disturbances that can eventually lead to blood-ocular barrier disruption.

Increased RPE cell apoptosis critically disrupts immunological homeostasis in the eye. As T-cell lines may adhere, in the present study, we confirmed the absence of T cells by microscopy after three passages of irradiated T cells. We then counted HRPEpiCs to confirm apoptosis. To comprehensively examine the effect of anti-VEGF treatment on RPE cells co-cultured with HTLV-1–infected T cells, the HRPEpiC line ARPE19 and primary HRPEpiCs were selected for cell counting. Proliferation of HTLV-1–infected RPE cells should be observed if anti-VEGF treatment promotes their growth. However, anti-VEGF treatment did not affect the number of ARPE19 cells or HRPEpiCs (**Figure 7**). These findings suggest that anti-VEGF treatment has no effect on RPE cell growth during HTLV-1 infection.

The results of this study should be interpreted in light of some limitations. First, despite lethally irradiating co-cultured T-cell lines, it was not possible to rule out the possibility that irradiated T cells may have fused to HRPEpiC/ARPE19 cells before elimination. The present study was designed as a preliminary *in vitro* analysis because we were unable to accurately reproduce all aspects of *in vivo* conditions. In addition to the clinical response of aflibercept in the eye, it is important to evaluate the therapeutic efficacy of anti-VEGF treatment in patients who are suffering from inflammatory diseases related to the presence of HTLV-1. In conjunction with basic research, long-term clinical tracking investigations of patients treated with anti-VEGF treatment might also be required.

Second, we used only one dose (0.5 mg/mL) of aflibercept, based on previous experiments that showed that aflibercept does not cause changes in cell morphology, apoptosis, or permanent reductions in cell viability, cell density, or proliferation of ARPE19 cells at clinical concentrations equivalent to those in the eye after vitreous injection (0.5 mg/mL) or at four times the clinical concentration (4 mg/mL) (53). Therefore, we used aflibercept in co-culture at a concentration equivalent to the clinical concentration to better mimic the intraocular environment of HTLV-1 carriers when treated with aflibercept. However, confirming the safety of aflibercept using *in vitro* experiments with various higher doses would be important. Further variable-dose experiments are needed to more clearly confirm the safety of aflibercept.

## Conclusion

In our study, we demonstrated that anti-VEGF treatment does not affect the production of activated NF-κB, inflammatory cytokines, or chemokines and does not increase the PVL or proliferation of RPE cells during HTLV-1 infection *in vitro*. In an *in vitro* setting simulating ATL, anti-VEGF treatment did not induce the production of activated NF-κB or chemokines or increase the PVL or cell proliferation, but did increase the production of the inflammatory cytokine IL-6 in the RPE. In addition, anti-VEGF treatment did not induce an increase in PVL in HTLV-1–infected T cells. The results indicate that for HTLV-1–infected individuals, intraocular anti-VEGF treatment would not exacerbate HTLV-1–related inflammation and thus appears to be safe, as far as this preliminary *in vitro* assessment is concerned.

## Data availability statement

The raw data supporting the conclusions of this article will be made available by the authors, without undue reservation.

## Author contributions

YZ performed the experiments and wrote the draft of the manuscript. KK designed the experiments, analyzed the data, wrote the manuscript, and obtained funding. HK-K, JZ, and MY performed the experiments. KO-M contributed to the analysis and interpretation of data and assisted in the preparation of the manuscript. All authors critically reviewed and approved the final manuscript. All authors contributed to the article and approved the submitted version.
